# Why Is There Medical Inertia and Nihilism to Celiac Disease? Comment on Pivetta et al. In Elderly Anemic Patients without Endoscopic Signs of Bleeding Are Duodenal Biopsies Always Necessary to Rule out Celiac Disease? *Diagnostics* 2022, *12*, 678

**DOI:** 10.3390/diagnostics12071510

**Published:** 2022-06-21

**Authors:** Emily A. Greenaway, Suneil A. Raju, David S. Sanders

**Affiliations:** Academic Department of Gastroenterology, Sheffield Teaching Hospital, Sheffield S10 2JF, UK; suneil.raju@nhs.net (S.A.R.); david.sanders1@nhs.net (D.S.S.)

We read, with interest, the paper entitled “In Elderly Anemic Patients without Endoscopic Signs of Bleeding Are Duodenal Biopsies Always Necessary to Rule Out Celiac Disease?” [1].

The study evaluated the findings of duodenal biopsies in 469 adult patients with anaemia (females n = 315 (67%); median age 54.7 years (18–93)) and the association of histological pathology with increasing age. It was found that patients with histopathological findings on duodenal biopsies were younger than those who had a normal duodenal mucosa (median 47.5 years vs. median 62 years, respectively, *p* = 0.0005). Secondly, patients with histological duodenal findings (celiac disease (CD) and others) were more likely to have concurrent macroscopic endoscopy findings (22.5% vs. 6.7%, *p* = 0.0014). The study also found a significant decreasing trend in histopathological findings with increasing age (*p* = 0.001). Anaemic patients with a macroscopically normal mucosa and increasing age were 3–4 times as likely to have a normal duodenal histology than those who were younger and displayed denting and decreased duodenal folds during endoscopy [1].

The authors concluded that, as histopathological findings were so infrequent in elderly anaemic patients—only one patient over the age of 70 was found to have CD in this study—the practice of routine duodenal biopsies in elderly anaemic patients is questionable.

In this study, the investigators divided those with duodenal intraepithelial lymphocytosis into a separate group; however, other studies have shown that 16% of these patients have CD upon a further extensive investigation [2]. Therefore, this inaccurately reduces the number of patients given a diagnosis of CD and is a missed opportunity.

We would strongly assert that biopsies should be taken and provide four key reasons from the published literature. Firstly, the chance to improve patients’ symptoms should be taken without delay. CD affects 1 in 100 individuals [3]. Currently, in the UK and internationally, the majority of cases remain undiagnosed [4]. There is also a recognised mean delay in the diagnosis of all CD patients between the ages of 9.7 and 12.8 years [5]. This is particularly apparent in the elderly population, where the delay in diagnosis can be up to 17 years [6]. We recently demonstrated in a multicentre study (n = 1423) that 12.4% of patients with newly diagnosed CD had a nondiagnostic gastroscopy (where no biopsies were taken) in the 5 years prior to their diagnosis [5].

Secondly, there is a significant mortality and morbidity associated with undiagnosed CD. Undiagnosed patients continue to consume gluten and experience the consequences of ongoing, unrecognised inflammation, particularly the elderly CD population which is at an even higher risk of CD-related malignancy and low-impact bone fractures [7,8].

Thirdly, the most recent published cost-effective study using a quality-of-life-adjusted model clearly demonstrated that undertaking a duodenal biopsy is cost-effective irrespective of age [9].

Finally, the recommended approach in that this study assumes that an elderly patient would not benefit or wish to know whether they have CD. This takes away patient choice and the opportunity to make an informed decision. A recent review of the literature in elderly patients with CD suggested that adherence to a gluten-free diet (GFD) and an improvement of symptoms can be achieved in up to 90% of individuals [8].

Why would the medical community behave in this paternalistic manner? We speculate that the societal negative connotations of the words ‘gluten free’ and the perceived lack of clinician input in the management of CD with a GFD results in medical inertia or nihilism towards CD [5]. Perhaps if there was a drug therapy for CD, this would not be the case? We fear that the discouragement of duodenal biopsies in certain patient groups is reinforcing the current inertia amongst clinicians, despite the evidence base we have presented.

In summary, we recommend following the current UK and US guidelines [10,11,12], as it is only through carrying this out that we afford all patients with unrecognised CD an opportunity to be diagnosed and make an informed decision for themselves.
